# Supplementary material for: A high-throughput screen indicates gemcitabine and JAK inhibitors may be useful for treating pediatric AML
Source: Nat Commun. 2019 May 16;10:2189. doi: 10.1038/s41467-019-09917-0 (PMC6522510; doi:10.1038/s41467-019-09917-0)
Supplement: Supplementary file 1 — Supplementary Information [file 41467_2019_9917_MOESM1_ESM.pdf]

**Supplementary Information**

**A high-throughput screen indicates gemcitabine and JAK inhibitors may be useful for treating pediatric AML** (Drenberg et al.)

## Supplementary Tables

### Supplementary Table 1. Characteristics of cell lines evaluated in high-throughput drug screen.

| Cell line | Subtype | Clinical Data | Genomic Alterations/Other features |
|---|---|---|---|
| CHRF288-11 | M7 | 2 yo, M, during treatment | JAK2 T875N; NUP98-KDM5A; HOXB6-HOXB3 |
| CMK | M7 | 10 mo, M, relapse | down syndrome; JAK3 A572V; TP53-FXR2; ADNP-NSF |
| CMS | M7 | 6 mo, F, relapse | CBFA2T3-GLIS2 |
| M07e | M7 | 6 mo, F, diagnosis | CBFA2T3-GLIS2; IL-3 dependent in culture |
| ML-2 | M4 | 26 yo, M, diagnosis | TALDO1-EPS8L2; KMT2A-MLLT4 |
| U937 | M5 | 37 yo, M, refractory | MLLT10-PICALM; PTEN null |
| MV4-11 | M5 | 10 yo, M, diagnosis | FLT3-ITD; AFF1-KMT2A |
| MOLM-13 | M5a | 20 yo, M, relapse | FLT3-ITD; KMT2A-MLL3T |

F, female, M, male; mo, month old; yo, year old; genomic alterations determined by RNA-seq

## Supplementary Table 2. Total number of hits in primary high-throughput screen.

| Cell line | Total Hits (>50) | Selective Hits (80/20) |
|---|---|---|
| CHRF28811 | 617 | 4 |
| CMK | 624 | 6 |
| CMS | 376 | 1 |
| ML2 | 334 | 0 |
| MO7E | 368 | 1 |
| MOLM13 | 571 | 5 |
| MV411 | 576 | 2 |
| U937 | 409 | 2 |

80/20, activity >80% in specific cell line and activity <20% in all other cell lines

## Supplementary Table 3. Activity of compounds in pediatric AML blast samples.

| Compounds | MOA | MLLr $IC_{50}$ (nM) | FLT3-ITD+ $IC_{50}$ (nM) | AMKL $IC_{50}$ (nM) |
|---|---|---|---|---|
| Sorafenib | Multi-kinase | 3000 - >40K | 700 - >30K | NE |
| Trametinib | MEK | 1.6 - 1472 | 130 - 5309 | NE |
| Ibrutinib | BTK | >20K | 5095 - >10K | NE |
| Palbociclib | CDK | 1114 - 3996 | 979 - 5257 | 5170 |
| Alisertib | Aurora A | 105 - 4315 | 8.8 - 3189 | 441 |
| Volasertib | PLK | 62 - 482 | 4.6 - 931 | 353 |
| Pictilisib | pan-PI3K | 536 - >50K | 337 - >50K | NE |
| MK-1775 | Checkpoint | 258 - >10K | 294 - >10K | >10K |
| Talazoparib | Checkpoint | 871 - >10K | 1981 - 2460 | NE |
| Carfilzomib | Proteasome | 4.3 - 53 | 6 - 62 | 54 |
| Bortezomib | Proteasome | 1.6 - 5 | 2.4 - 2.7 | 20 |
| RG7112 | MDM2 | 149 - 845 | 77 - 952 | NE |
| Venetoclax | Apoptosis | 46 - 1049 | 3.2 - 16 | NE |
| Vorinostat | HDAC | 711 - 1195 | 304 - 2886 | 637 |
| Panobinostat | HDAC | 0.9- 3.3 | 0.7 - 2.6 | 14 |
| Romidepsin | HDAC | 1.6 - 6 | 3.8 - 13 | 5 |
| Cabazitaxel | Microtubule | 3.4 - >20K | 12 - >50K | >50K |
| Gemcitabine | Anti-metabolite | 267 - 4233 | 102 - >10K | 108 |
| Cytarabine | Anti-metabolite | 122 - >8K | 60 - >5K | 54 |
| Artesunate | Anti-malarial | 3000 - >40K | 700 - >30K | NE |

MOA, mechanism of action; MLLr, MLL rearranged; FLT3-ITD+, FLT3 internal tandem duplication positive; AMKL, acute megakaryoblastic leukemia; NE, not evaluated

**Supplementary Table 4. Pharmacokinetic parameters of ruxolitinib.**

| Strain | Gender | $T_{1/2}$, h | $T_{max}$, h | $C_{max}$, µM | AUClast, µM*h |
|---|---|---|---|---|---|
| BoyJ | Female | 2.03 ± 1.10 | 0.25 | 18.1 ± 10.6 | 16.8 ± 10.8 |
| | Male | 2.64 ± 1.74 | 0.25 | 28.6 ± 10.6 | 17.9 ± 6.5 |
| NSG | Female | 1.26 ± 0.48 | 0.25 | 6.9 ± 1.7 | 4.8 ± 0.6 |
| | Male | 1.32 ± 0.74 | 0.25 | 5.5 ± 1.7 | 4.1 ± 0.6 |

## Supplementary Figures

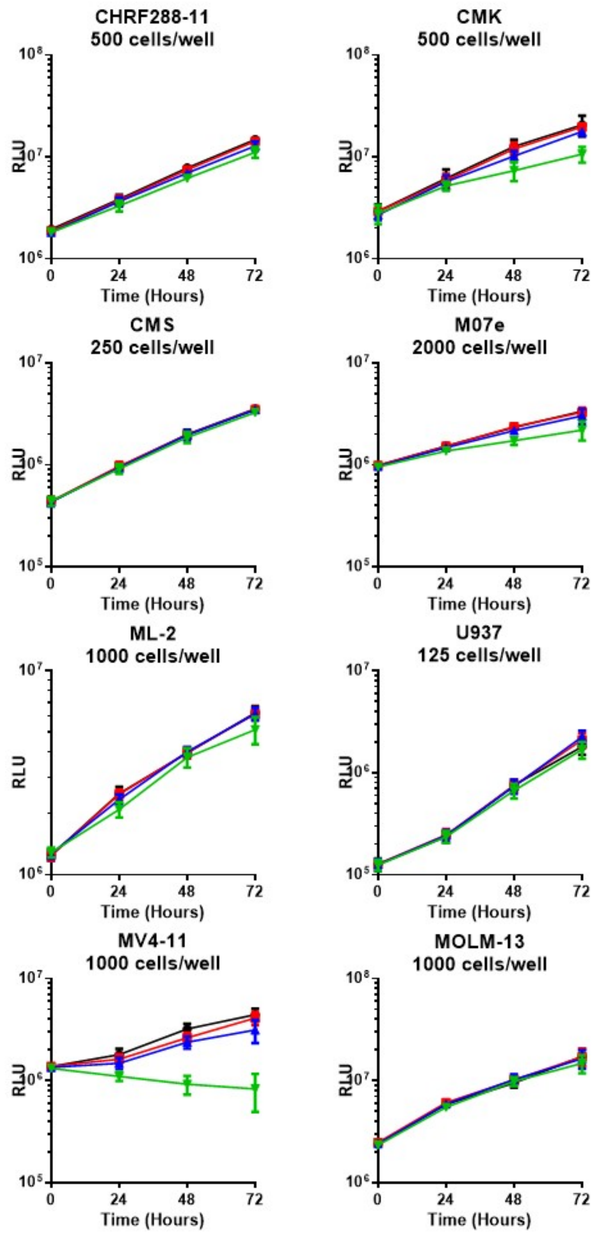


**Supplementary Figure 1. Validation of AML cell lines for high-throughput screening.** AML cell lines were seeded at a pre-determined density as indicated for each cell line based on growth rate and suitable for 96 hours in a 384-well plate. Increasing volumes of DMSO (0 nL, black; 10 nL, red; 50 nL, blue; 100 nL, green) were pintool transferred using an automation workstation at 24 hours after plating. Cell viability was determined using Cell Titer Glo at the indicated time points (0, 24, 48, and 72 hours). Data is reported as mean relative luminescence units (RLU) plus/minus standard deviation (N=96 samples per time point).

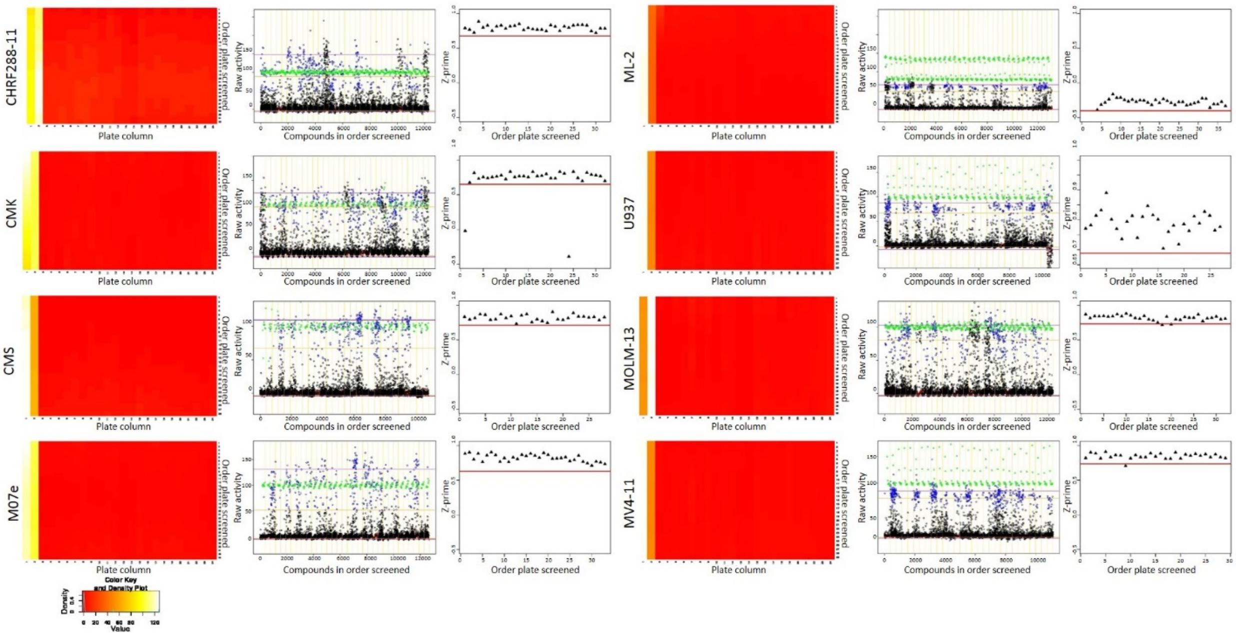


**Supplementary Figure 2. Quality control of high-throughput screening.** For each cell line we show, left panels: distribution of the reference compound (cycloheximide) effective concentration for each plate in the primary screen. The reference compound behaves similarly across all 8 cell lines and is highly reproducible from plate to plate. Center panels: Scatter plot of percent inhibition of positive control (green), negative control (red), and screened compounds (black). Right panels: Distribution of Z-prime values for each plate in the primary screen. All Z-prime values were >0.5 with the exception of plates 1 and 24 of the CMK cell line.

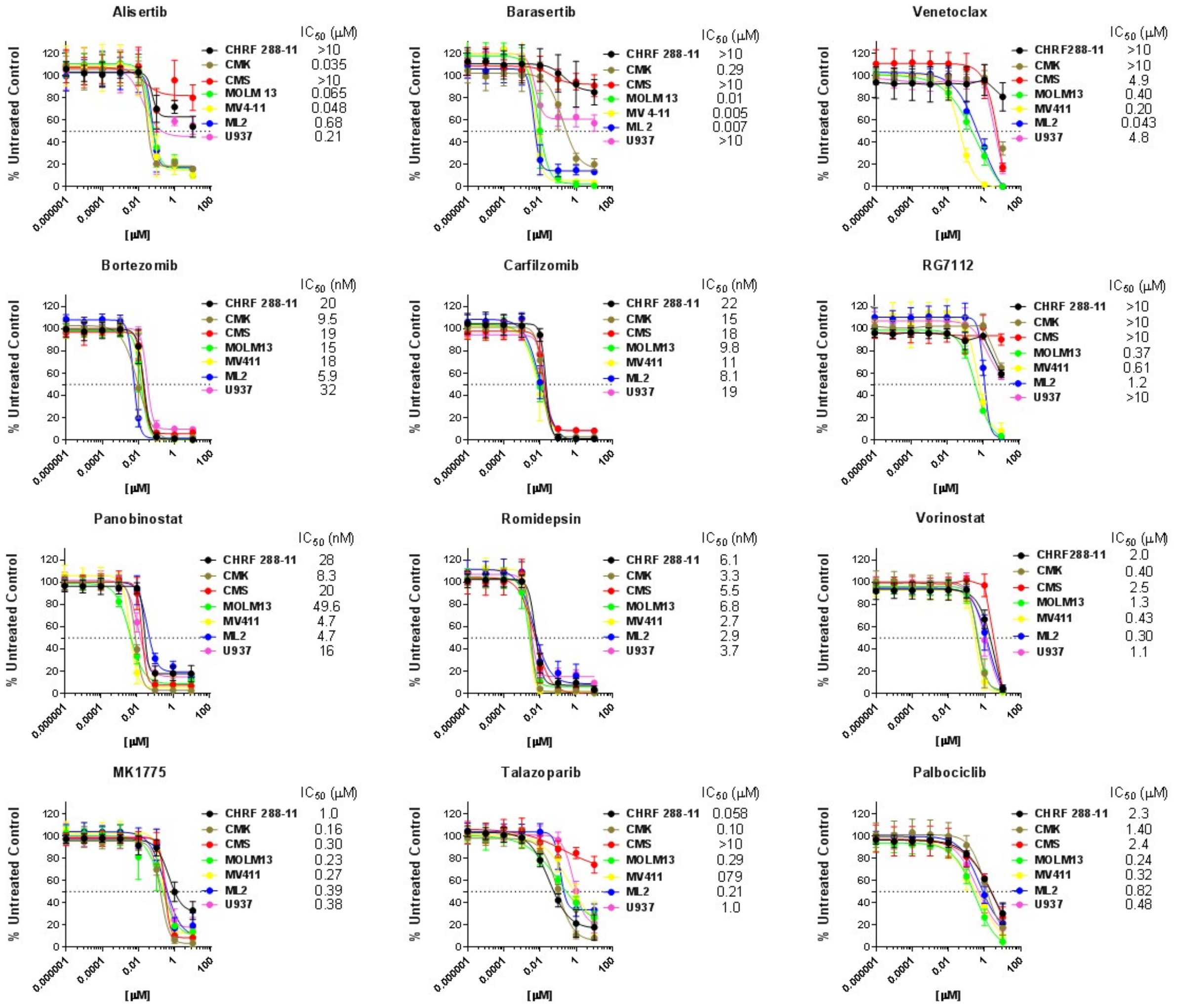


**Supplementary Figure 3. Validation of compounds from secondary screen.** Anti-leukemic activity of selected compounds (N=12) were determined in a low-throughput manner using a 96-well plate format; cells were exposed to increasing concentrations (0-10 µM; 1:10 dilution) and cell viability was determined at 72 hours using MTT (5 mg/mL). The half maximal inhibitory concentration (IC50, dotted line) was evaluated by nonlinear regression analysis using GraphPad Prism. Data are reported as percent untreated control and represented as mean plus/minus standard deviation of 3 independent experiments (N=18 per concentration).

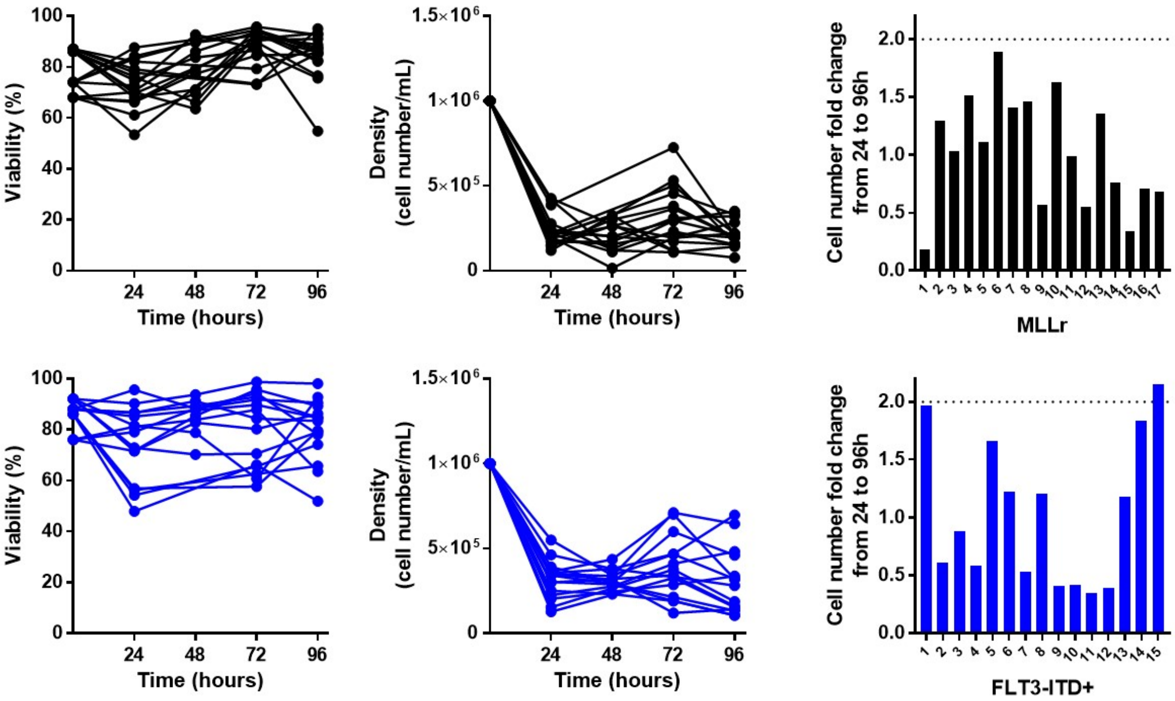


**Supplementary Figure 4. Monitoring of primary patient samples *ex vivo*.** Anti-leukemic activity of selected compounds were determined in a low-throughput manner using a 96-well plate format; MLL rearranged (MLLr, black) or FLT3-internal tandem duplication positive (FLT3-ITD+, blue) primary patient blasts from were co-cultured with human mesenchymal stromal cells then exposed to increasing concentrations of select compounds. Cell viability (left panels) and density (center panels) in untreated wells was determined at 0, 24, 48, 72, and 96 hours using acridine orange/propidium iodide with a Cellometer K2. Doubling of each patient sample was determined from 24 to 96 hours (right panels); data reported as the cell number fold change (doubling indicated by dotted line).

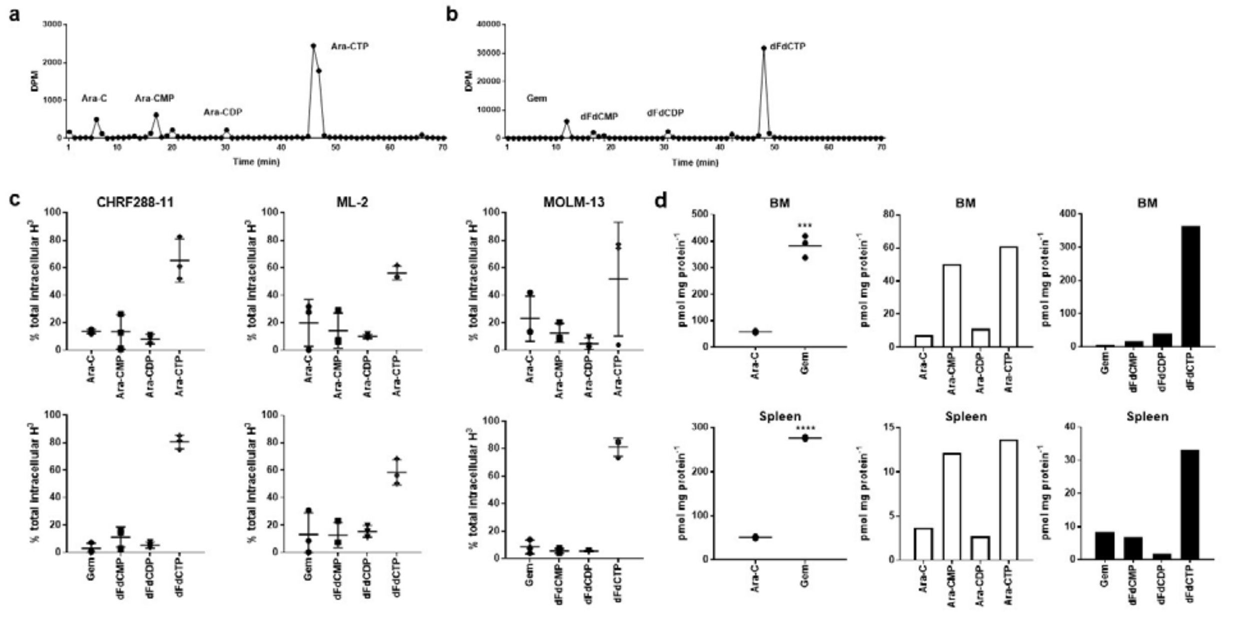


**Supplementary Figure 5. Accumulation of cytarabine and gemcitabine.** Accumulation assays (2 h) in AML cells were performed using a mixture (1µM) of unlabeled and [$^3$H]-cytarabine (Ara-C) or gemcitabine (Gem). Intracellular Ara-C, Gem, and phosphorylated metabolites (Ara-CMP, Ara-C monophosphate; Ara-CDP, Ara-C diphosphate; Ara-CTP, Ara-C triphosphate; dFdCMP, Gem monophosphate; dFdCDP, Gem diphosphate; dFdCTP; Gem triphosphate) levels were determined by liquid scintillation counting and normalized to protein levels. Representative chromatogram for (a) Ara-C and (b) Gem using HPLC coupled with liquid scintillation counting; data shown as disintegrations per minute (DPM) over complete run time. (c) Percentage of total intracellular [$^3$H]-labelled parent drug and metabolites (Ara-C, top panel; Gem, bottom panel); data are presented as mean plus/minus (±) standard deviation (SD) of three independent experiments (N=9). (d) Accumulation was determined in cell lysate of bone marrow (top panels) and spleen (bottom panels) from a treatment naive $Mll^{PTD/wt}$:$Flt3^{ITD/ITD}$ double knock-in primary transplant recipient. Results for total accumulation (d, left panels) are shown as pmol/mg protein and presented as mean ± SD from one independent experiment performed in triplicate. Student's t-test; ***P<0.0005; ****P<0.0001

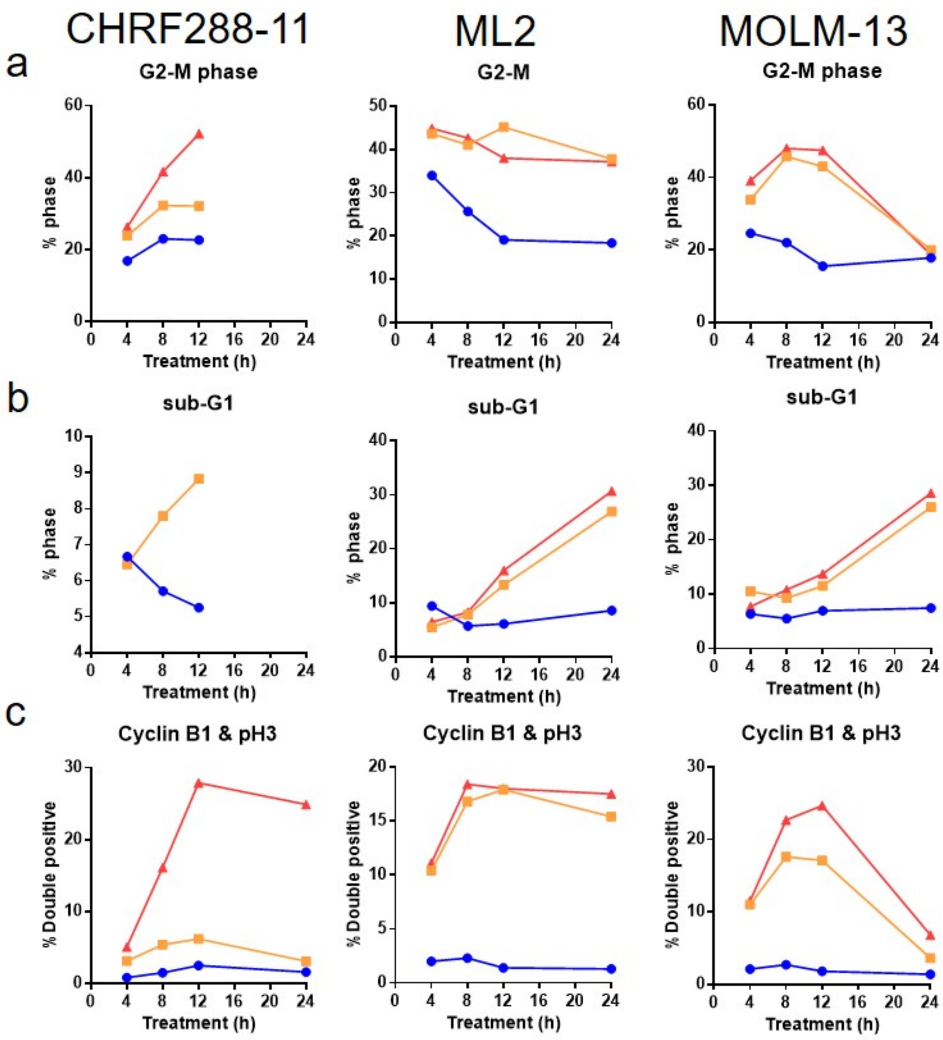


**Supplementary Figure 6. Cabazitaxel induces G2/M cell cycle arrest.** Asynchronous CHRF288-11 (left), ML-2 (center), and MOLM-13 (right) were treated with 5 nM (orange) or 50 nM (red) cabazitaxel; DMSO (blue) was the control agent. Cell cycle distribution was assessed by propidium iodide staining at 4, 8, 12, and 24 h; the percentage of cells in (a) G2/M and (b) sub-G1 over time are shown. (c) Induction of mitosis was determined by biparametric flow cytometry analysis, using phospho-histone H3-FITC and cyclin B-APC staining; the percentage of double-positive cells over time are shown from one independent experiment.

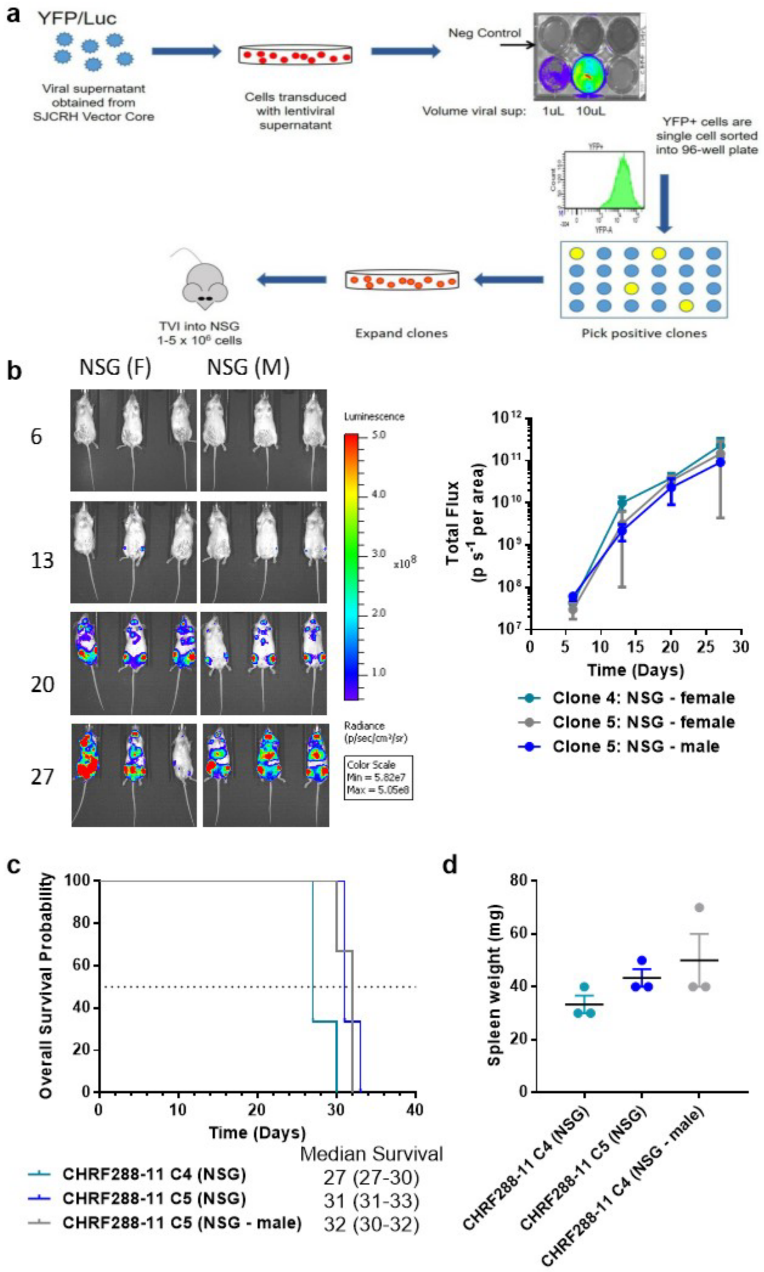


**Supplementary Figure 7. Establishment of a CHRF288-11 cell line xenograft model.** (a) Schematic of workflow used for development of a CHRF288-11 cell line xenograft model. AML cell line is transduced with lentiviral supernatant with YFP/luciferase containing particles. After 24-48 h cells are assessed for luciferase activity by adding luciferase substrate D-luciferin firefly potassium salt (20 µL of 47.2mM stock) to each well; bioluminescence is measured using a Xenogen in vivo imaging system. Cells are then subjected to single cell sort using YFP, whereby one cell is flow sorted into each well of a 96-well plate. Individual clones are expanded then tail vein injected into female and male 8-12 week old NSG mice. (b) Engraftment is monitored by serial weekly bioluminescence imaging using a Xenogen IVIS-200 and Living Image software; representative image (left panel) and quantification (right panel) mean plus/minus standard deviation (N=3). (c) Kaplan-Meier analysis of animal survival. (d) Spleen weight (mg) at time of sacrifice.

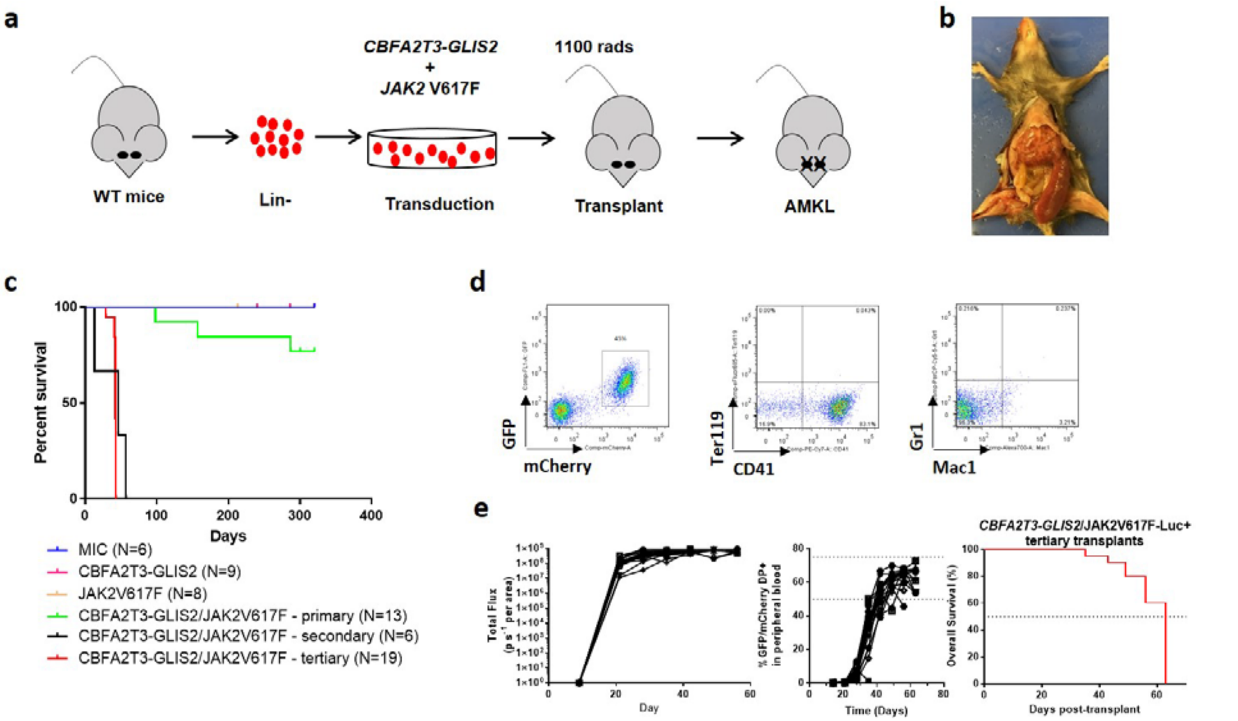


**Supplementary Figure 8. Transduction/transplantation model of AMKL.** (a) Schematic for *in vivo* model. Murine bone marrow is lineage depleted (Lin-) and transduced with a control retroviral construct (MIC, blue), CBFA2T3-GLIS2 only (purple), JAK2 V617F only (tan), and CBFA2T3-GLIS2 plus JAK2 V617F (CBFA2T3-GLIS2/JAK2V617F) followed by injection into lethally irradiated primary recipients (green) and sublethally irradiated secondary (black) and tertiary recipients (red). (b) CBFA2T3-GLIS2/JAK2V617F primary transplant recipients show an enlarged spleen. (c) Kaplan-Meier analysis of animal survival. (d) Flow cytometry analysis of bone marrow cells harvested from moribund primary recipients, CBFA2T3-GLIS2 (mCherry), JAK2V617F (GFP) double positive cells were gated and analyzed with lineage markers as indicated. Representative analysis are shown. AMKL blasts isolated from secondary transplants were transduced with a luciferase (Luc)-BFP retroviral construct, mCherry/GFP/BFP-triple positive cells were injected into sublethally irradiated tertiary recipients. (e) Engraftment was monitored by serial weekly bioluminescence imaging (left) and detection of mCherry/GFP-double positive (DP) cells in the peripheral blood (center); Kaplan-Meier analysis of animal survival (right).

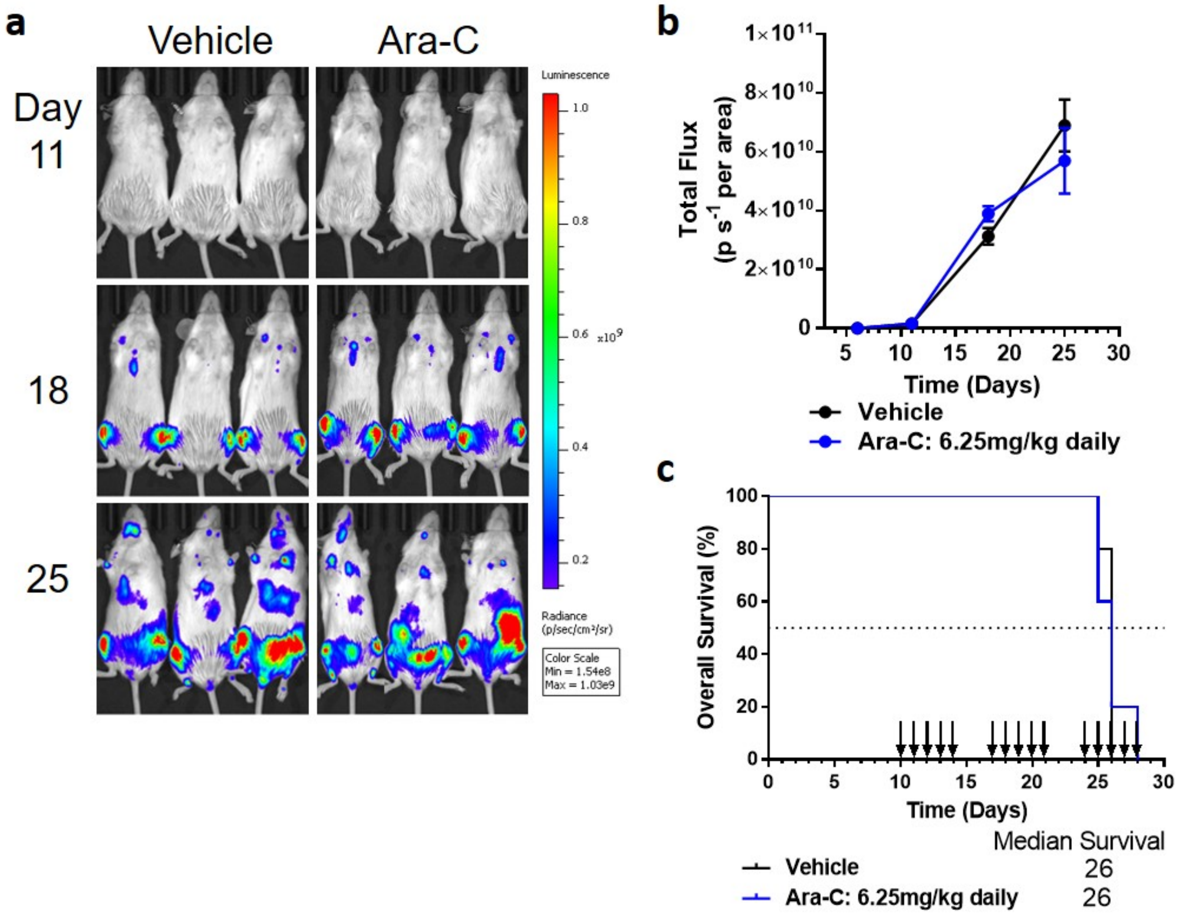


**Supplementary Figure 9. *In vivo* activity of cytarabine.** Female NSG mice engrafted with CHRF288-11-Luc/YFP+ cells were treated with vehicle (black) or cytarabine (Ara-C, blue) 6.25mg/kg once daily for 5 days (Monday to Friday) for 3 consecutive weeks beginning on day 10. (a) Serial bioluminescence images of representative mice from each treatment group and (b) quantification of bioluminescent signal mean plus/minus standard error (N=5 per treatment group) were acquired using a Xenogen IVIS-200 and Living Image software. (c) Kaplan-Meier analysis of animal survival; arrows indicate treatment days.

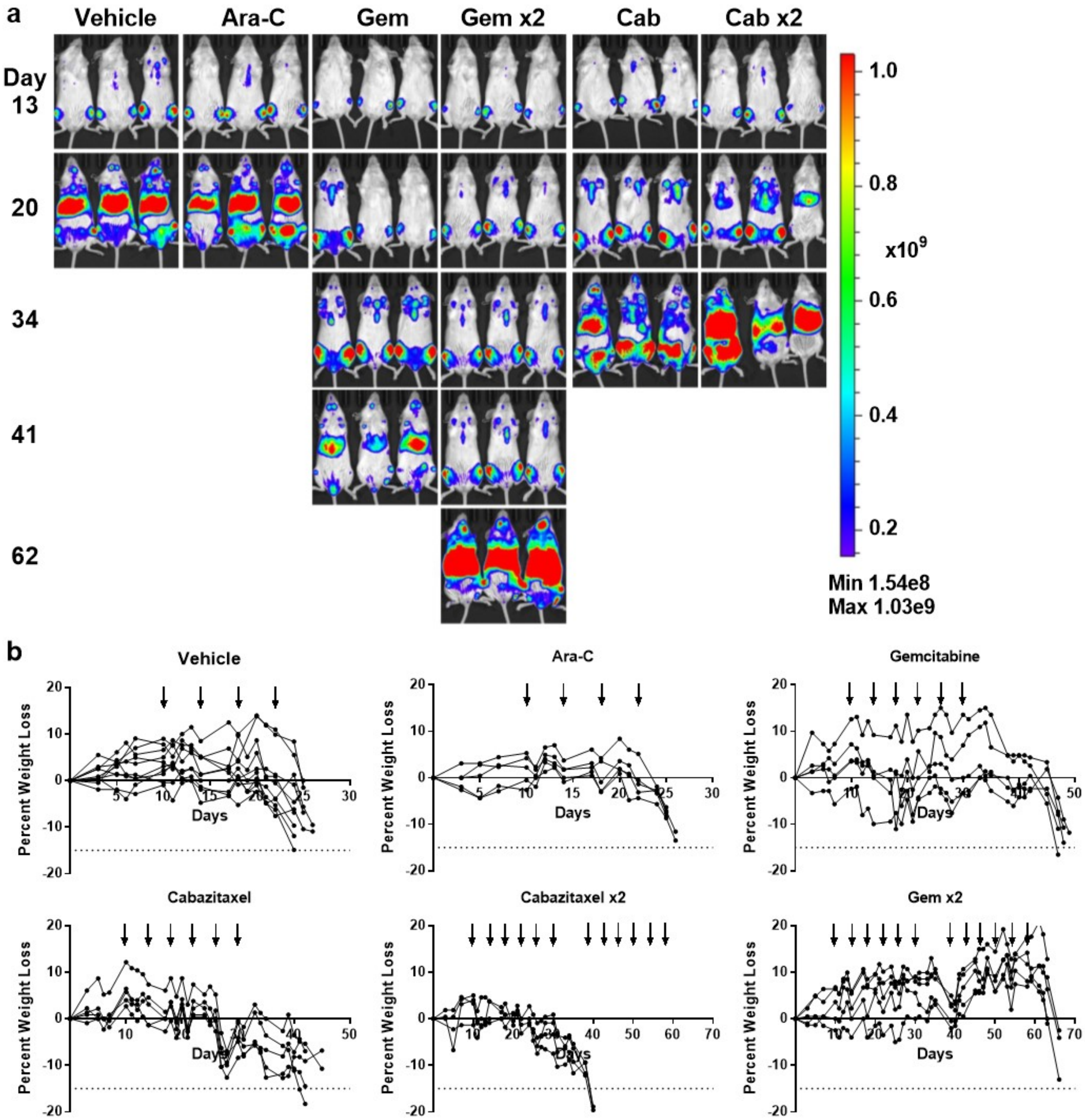


**Supplementary Figure 10. Monitoring tumor progression in CHRF288-11 xenograft.** Female NSG mice engrafted with CHRF288-11-YFP/Luc-positive cells were treated with vehicle, 50 $mgkg^{-1}$ cytarabine (Ara-C), 50$mgkg^{-1}$ gemcitabine (Gem), or 5 $mgkg^{-1}$ cabazitaxel (Cab). All treatment were administered by intraperitoneal injection starting on day 10 and continued once every 4 days for 3 weeks for one cycle; alternatively, after one week off treatment a second 3 week cycle was administered (two cycles, x2). (a) Serial bioluminescence images of representative mice treated as indicated; acquired using a Xenogen IVIS-200 and Living Image software. (b) Daily weight of treated mice; data reported as a percent weight loss from day 0. Arrow indicate treatment days.

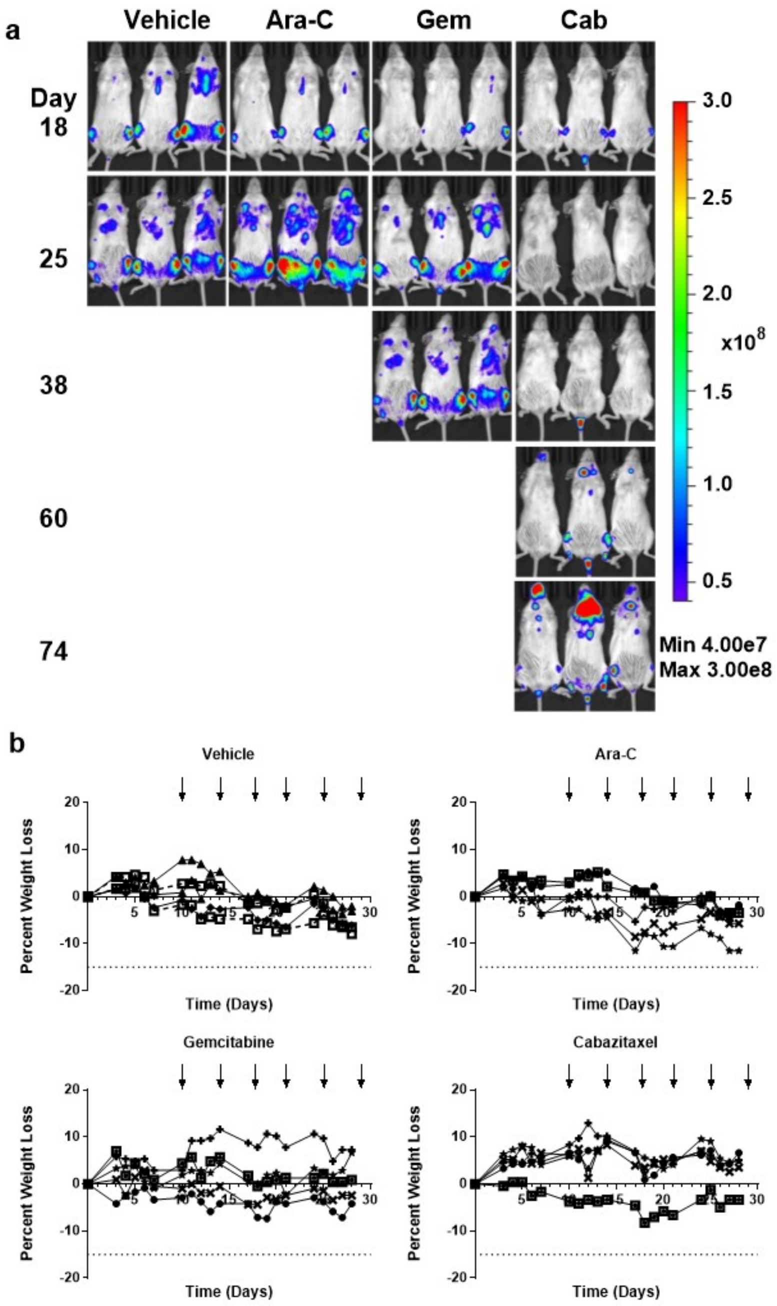


**Supplementary Figure 11. Monitoring tumor progression in ML-2 xenograft.** Female NSG mice engrafted with ML-2-YFP/Luc-positive cells were treated with vehicle, 50 mgkg$^{-1}$ cytarabine (Ara-C), gemcitabine 50mgkg$^{-1}$ (Gem), or cabazitaxel 5 mgkg$^{-1}$ (Cab) every 4 days. All treatments were administered by intraperitoneal injection starting on day 17 and continued once every 4 days for 3 weeks for two cycles. (a) Serial bioluminescence images of representative mice treated with the indicated drug(s); acquired using a Xenogen IVIS-200 and Living Image software. (b) Daily weight of treated mice; data reported as a percent weight loss from day 0. Arrow indicate treatment days.

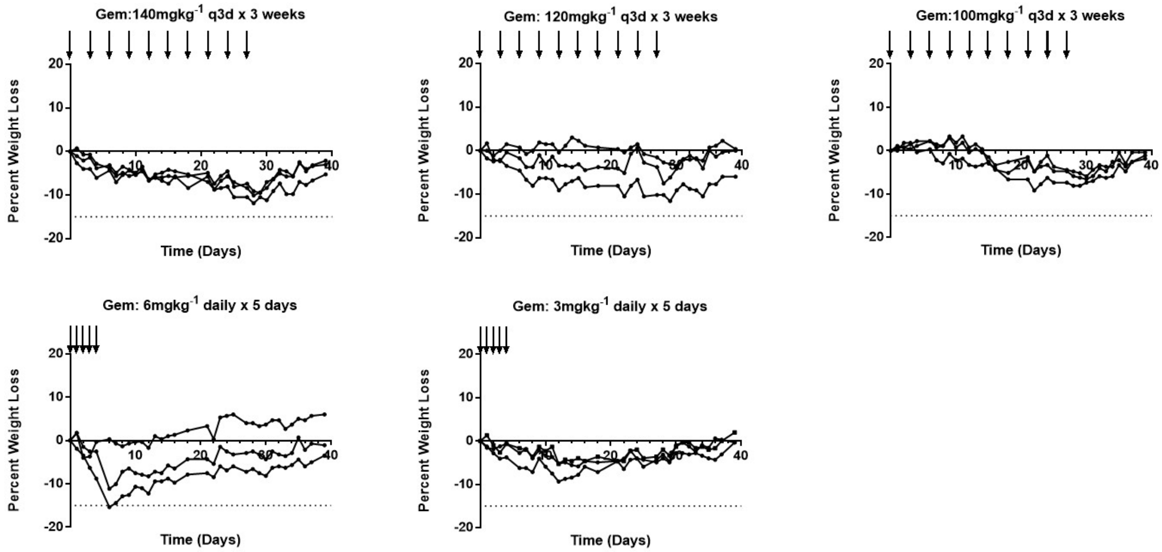


**Supplementary Figure 12. Establish maximum tolerated dose of gemcitabine.** Non-tumor bearing female BoyJ mice were treated on an intermittent schedule every 3 days for 3 weeks with 100, 120, or 140 mgkg$^{-1}$ gemcitabine (Gem) or treated on a daily schedule once a day for 5 days with 3 or 6 mgkg$^{-1}$ Gem. Daily weight of treated mice; data reported as a percent weight loss from day 0. Arrow indicate treatment days.

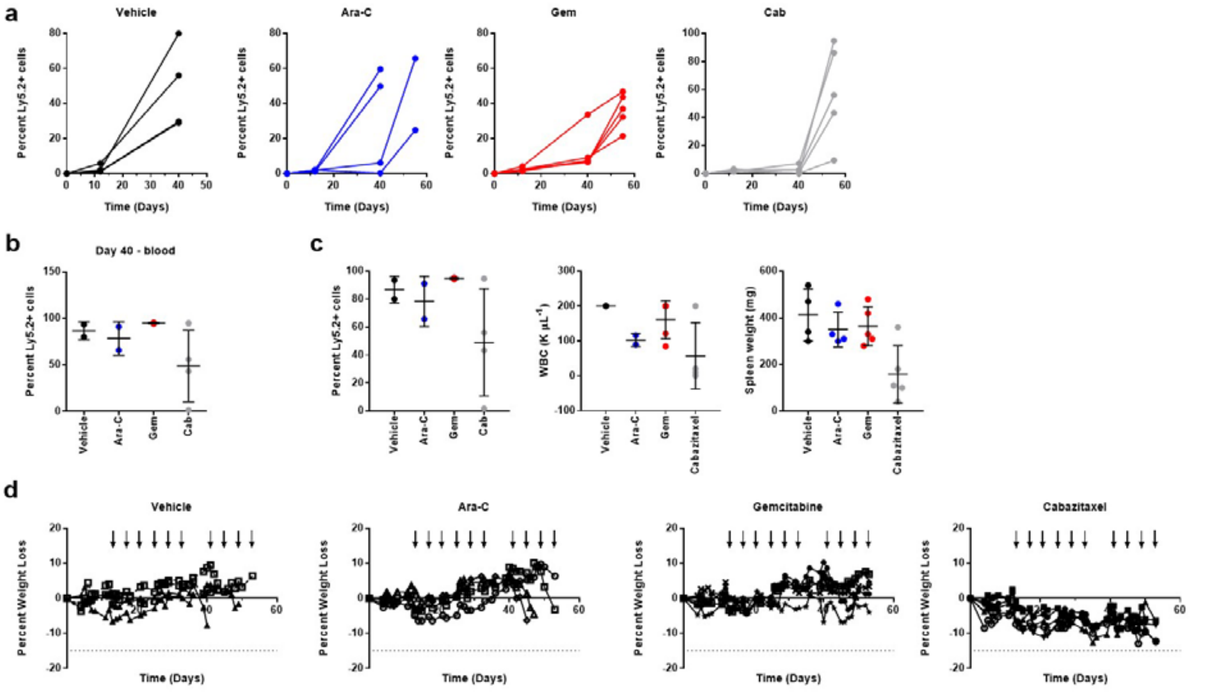


**Supplementary Figure 13. Monitoring tumor progression in Mll$^{PTD/wt}$:Flt3$^{ITD/ITD}$ primary transplants.** Whole bone marrow and spleen cells isolated from a single leukemic Ly5.2+ double knock-in mouse were pooled and intravenously injected into the tail vein of sublethally irradiated Ly5.1+ syngeneic C57BL/6 mice. Mice were randomized to receive vehicle, 50 mgkg$^{-1}$ cytarabine (Ara-C), gemcitabine 50mgkg$^{-1}$ (Gem), or cabazitaxel 5 mgkg$^{-1}$ (Cab) every 4 days. Blood was collected from the submandibular vein of mice every two weeks throughout the entire study. (a) Percentage of Ly5.2+ cells in peripheral blood of individual mice per treatment group (cytarabine, Ara-C; gemcitabine, Gem; cabazitaxel, cab) over time course of study. (b) Mean percentage of Ly5.2+ cells plus/minus (±) standard deviation (SD) per treatment group on day 40 of study; vehicle (N=2 mice); Ara-C (N=2, mice); Gem (N=5 mice); Cab (N=4 mice). (c) Mean percentage of Ly5.2+ cells ± SD in peripheral blood (left), WBC count (center), and spleen weight (right) per treatment group at sacrifice. (d) Daily weights of treated mice; data reported as a percent weight loss from day 0. Arrow indicate treatment days.

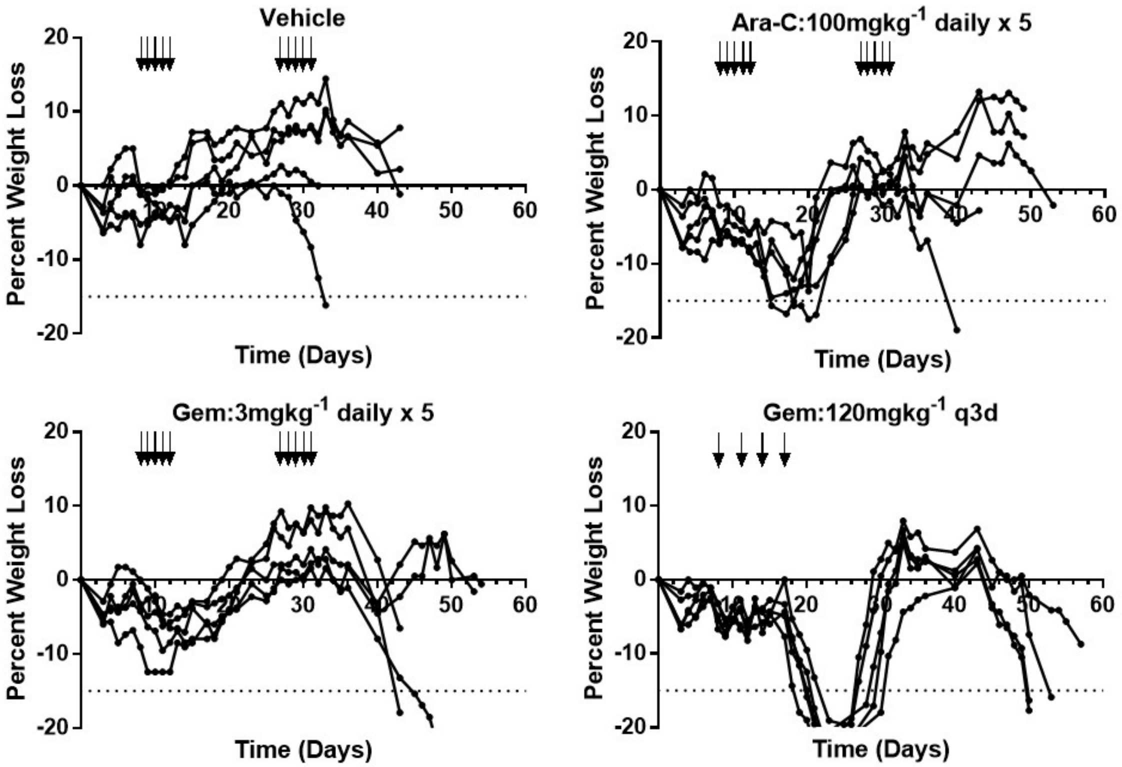


**Supplementary Figure 14. Tolerability of gemcitabine in Mll$^{PTD/wt}$:Flt3$^{ITD/ITD}$ secondary transplants.** Female BoyJ mice were sublethally irradiated on day 0, after 4 hours Ly5.2+ cells isolated from the bone marrow and spleen of primary transplants were intravenously injected into the tail vein of secondary transplants. Mice were randomized on day 8 to receive vehicle, 100mgkg$^{-1}$ daily by 5 days cytarabine (Ara-C daily x 5), 3mgkg$^{-1}$ daily by 5 days or 120mgkg$^{-1}$ once every 3 days gemcitabine (Gem daily x 5 and q3d, respectively). Daily weights of secondary transplants; data reported as a percent weight loss from day 0. Arrow indicate treatment days.

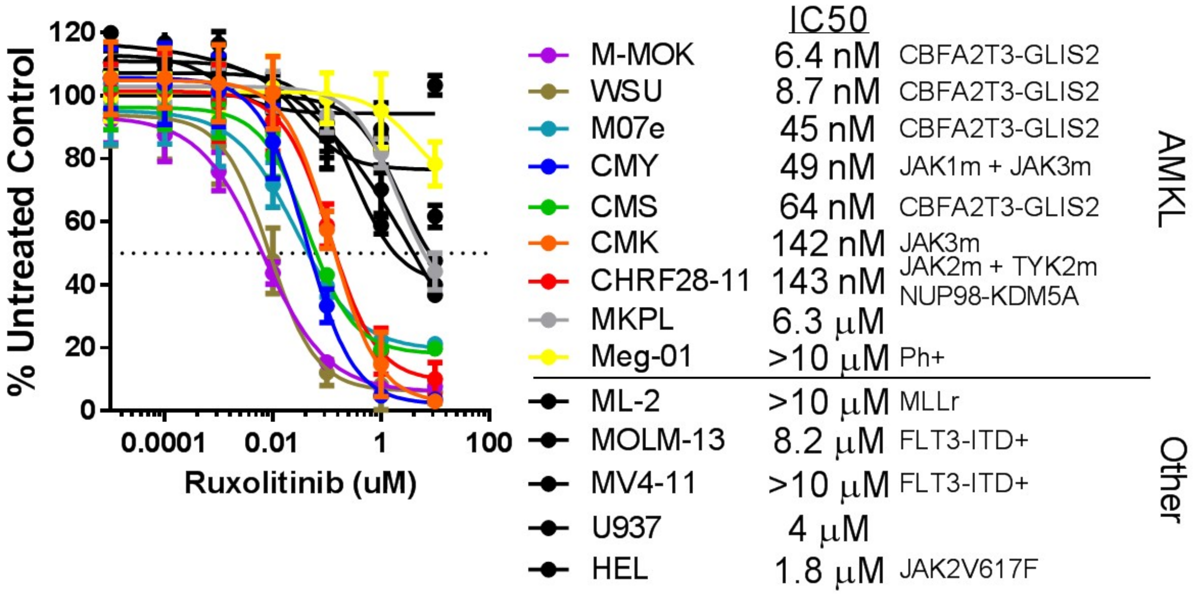


**Supplementary Figure 15. Selective activity of JAK inhibitors for AMKL.** A panel of AML cell lines were treated with increasing concentrations of vehicle or ruxolitinib for 72 h, and cell viability was measured using Cell Titer Glo. The half maximal inhibitory concentration (IC50) was evaluated by nonlinear regression analysis using GraphPad Prism. Data are reported as percent untreated control and represented as mean plus/minus standard deviation of 3 independent experiments (N=18 per concentration). JAK1m, JAK1 mutated; JAK3m, JAK3 mutated; TYK2m, TYK2 mutated; Ph+, Philadelphia chromosome positive; MLLr, MLL rearranged; FLT3-ITD+, FLT3-internal tandem duplication positive

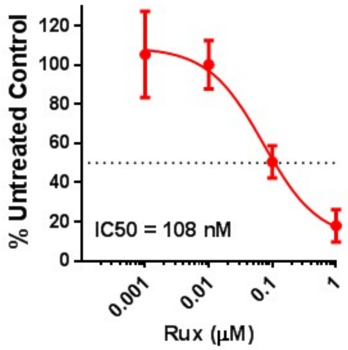


**Supplementary Figure 16. Activity of ruxolitinib in primary murine blasts.** Leukemic blasts were isolated from the bone marrow and spleen of CBFA2T3-GLIS2/JAK2V617F tertiary transplant recipients. Cells were plated in a 96-well plate and treated with increasing concentrations of ruxolitinib (Rux). Cell viability was determined at 72h using Cell Titer Glo. The half maximal inhibitory concentration (IC50, dotted line) was evaluated by nonlinear regression analysis using GraphPad Prism. Data are reported as percent untreated control and represented as mean plus/minus standard deviation (N=16 per concentration).
