# Supplementary material for: A high-throughput screen indicates gemcitabine and JAK inhibitors may be useful for treating pediatric AML
Source: Nat Commun. 2019 May 16;10:2189. doi: 10.1038/s41467-019-09917-0 (PMC6522510; doi:10.1038/s41467-019-09917-0)
Supplement: Supplementary file 3 — Description of Additional Supplementary Files [file 41467_2019_9917_MOESM3_ESM.pdf]

Description of Additional Supplementary Files

File Name: Supplementary Data 1
Description: Activity of compounds in primary high-throughput screen. Data are reported as percent inhibition per compound for each cell line relative to positive control

File Name: Supplementary Data 2
Description: Activity of compounds in secondary high-throughput screen. Data are reported as average effective concentration per compound for each cell line.
